# Treating and Preventing Influenza in Aged Care Facilities: A Cluster Randomised Controlled Trial

**DOI:** 10.1371/journal.pone.0046509

**Published:** 2012-10-17

**Authors:** Robert Booy, Richard I. Lindley, Dominic E. Dwyer, Jiehui K. Yin, Leon G. Heron, Cameron R. M. Moffatt, Clayton K. Chiu, Alexander E. Rosewell, Anna S. Dean, Timothy Dobbins, David J. Philp, Zhanhai Gao, C. Raina MacIntyre

**Affiliations:** 1 National Centre for Immunisation Research and Surveillance of Vaccine Preventable Diseases, The University of Sydney, Sydney, New South Wales, Australia; 2 Discipline of Medicine, The University of Sydney and The George Institute for Global Health, Sydney, New South Wales, Australia; 3 Centre for Infectious Diseases and Microbiology Laboratory Services, Institute of Clinical Pathology and Medical Research, Westmead Hospital and Sydney Institute for Emerging Infections and Biosecurity, The University of Sydney, Sydney, New South Wales, Australia; 4 Cancer Epidemiology and Services Research, Sydney School of Public Health, University of Sydney, Sydney, New South Wales, Australia; 5 School of Public Health and Community Medicine, University of New South Wales, Sydney, New South Wales, Australia; 6 Faculty of Medicine, School of Public Health and Community Medicine, The University of New South Wales, Sydney, New South Wales, Australia; University of Ottawa, Canada

## Abstract

**Background:**

Influenza is an important cause of morbidity and mortality for frail older people. Whilst the antiviral drug oseltamivir (a neuraminidase inhibitor) is approved for treatment and prophylaxis of influenza during outbreaks, there have been no trials comparing treatment only (T) versus treatment and prophylaxis (T&P) in Aged Care Facilities (ACFs). Our objective was to compare a policy of T versus T&P for influenza outbreaks in ACFs.

**Methods and Findings:**

We performed a cluster randomised controlled trial in 16 ACFs, that followed a policy of either “T”—oseltamivir treatment (75 mg twice a day for 5 days)—or “T&P”—treatment and prophylaxis (75 mg once a day for 10 days) for influenza outbreaks over three years, in addition to enhanced surveillance. The primary outcome measure was the attack rate of influenza. Secondary outcomes measures were deaths, hospitalisation, pneumonia and adverse events. Laboratory testing was performed to identify the viral cause of influenza-like illness (ILI) outbreaks. The study period 30 June 2006 to 23 December 2008 included three southern hemisphere winters. During that time, influenza was confirmed as the cause of nine of the 23 ILI outbreaks that occurred amongst the 16 ACFs. The policy of T&P resulted in a significant reduction in the influenza attack rate amongst residents: 93/255 (36%) in residents in T facilities versus 91/397 (23%) in T&P facilities (p = 0.002). We observed a non-significant reduction in staff: 46/216 (21%) in T facilities versus 47/350 (13%) in T&P facilities (p = 0.5). There was a significant reduction in mean duration of outbreaks (T = 24 days, T&P = 11 days, p = 0.04). Deaths, hospitalisations and pneumonia were non-significantly reduced in the T&P allocated facilities. Drug adverse events were common but tolerated.

**Conclusion:**

Our trial lacked power but these results provide some support for a policy of “treatment and prophylaxis” with oseltamivir in controlling influenza outbreaks in ACFs.

**Trail Registration:**

Australian Clinical Trials Registry ACTRN12606000278538

## Introduction

Influenza is a cause of significant morbidity and mortality [1], with the highest hospitalisation rates in the very young and the very old. In developed countries, the majority of deaths attributable to influenza occur in people aged over 65 years, especially those over 80 years with pre-existing health problems and residents of Aged Care Facilities (ACFs) [1]. Attack rates of 20–40% may occur in ACF outbreaks and are associated with increased rates of hospitalisation and death [2], [3]. The elderly may also shed influenza virus at higher levels and for longer duration than younger adults [4], [5], [6]. As a result, transmission to others is more likely. In addition, accentuated transmission of influenza within closed institutions is a recognised problem, which results in significant morbidity and costs [7], [8], [9].

Immunisation against influenza is recommended for high-risk populations in Australia including all persons aged 65 years and over [10]. Vaccination is less effective in the elderly [11], and influenza outbreaks have been documented in highly vaccinated ACF populations, where the intensity of transmission appears to override vaccine-conferred immunity [2]. Thus, additional strategies such as the use of antiviral therapy need to be considered.

Although oseltamivir is effective for treating and preventing influenza [12], [13], strategies of oseltamivir use in outbreaks, namely treatment alone, or treatment together with prophylaxis, have never been compared head to head. As frail institutionalised elderly are disproportionately affected by influenza outbreaks (yet rarely included in randomised controlled trials [14]), we decided to evaluate the effectiveness of these two different strategies in a cluster-randomised controlled clinical trial. We chose to randomly assign the strategies by facility, rather than by individual, because influenza is a communicable disease that once introduced into a facility can be readily transmitted, and because the use of oseltamivir to treat patients in an outbreak may result in “herd protective effects” beyond an individual effect.

Our aim was to test the hypothesis that a policy of treatment and prophylaxis with oseltamivir would be a more effective than treatment only. Our results provide some support for this hypothesis.

## Methods

### Ethics statement

The study was approved by the Ethics Committees of The University of Sydney and The Children's Hospital at Westmead. As oseltamivir was used in a currently approved manner in Australia, the study did not come under the Guardianship Tribunal legislation for clinical trials.

The protocol for this trial and supporting CONSORT checklist are available as supporting information; see Checklist S1 and Protocol S1. The cluster randomised controlled trial was a collaboration between The University of Sydney and Moran Health Care Group, the largest private provider of aged care facilities in Australia. The 16 ACFs owned and managed by the Moran Health Care Group within the greater metropolitan region of Sydney were asked to participate and all agreed.

The two different management policies used in the cluster RCT were:

Treatment only (T): A policy of using oseltamivir for treatment of both residents and staff with test-confirmed influenza and also residents and staff with influenza-like illness (ILI) that were epidemiologically linked by being resident in, or working in, the same wing or floor of the ACF as a test-confirmed case of influenza.

Treatment and Prophylaxis (T&P): A policy of using oseltamivir for treatment of individuals as specified above PLUS prophylaxis of consenting individuals who were resident in, or working in the same wing or floor of the ACF as a test-confirmed case of influenza.

### Pre-specified outcomes

The primary pre-specified outcome was the attack rate of influenza in the “T” versus the “T&P” ACFs (confirmed, probable and possible influenza cases) in residents and staff of ACFs during outbreaks of influenza. Secondary outcomes were: case fatality rate within 4 weeks from onset of an outbreak; pneumonia incidence in subjects within 4 weeks from onset of influenza symptoms; hospital admission incidence within 4 weeks from onset of influenza symptoms; adverse events within 4 weeks of commencing oseltamivir; and outbreak duration (defined as the date of the onset of the first symptomatic resident in a confirmed influenza outbreak to the date of onset of the last case).

### Oseltamivir dosing regimens

#### *Treatment*

Oseltamivir 75 mg orally twice daily for 5 days was offered to all persons with influenza diagnosed within 48 hours from the onset of symptoms, by point-of-care test or laboratory testing, and persons who met the clinical definition for ILI and whose symptoms had duration of less than 48 hours when an outbreak of influenza infection was identified in their ACF.

#### *Prophylaxis*

Oseltamivir 75 mg orally once daily for 10 days was offered to all staff and resident of T&P ACFs when an influenza outbreak was identified in their ACF.

The oseltamivir dose interval was doubled for participants with known renal impairment (creatinine clearance 10 to 30 ml/min).

### Sample size

To obtain 80% power at 2-sided 5% significance level for detecting a significant difference of attack rate between the two interventions (T, T&P), and for an assumed 10% (or 15%) attack rate in the T arm and 3% (or 6%) in the T&P arm, a sample size of 8 clusters (ACFs) or 360 subjects per arm was required for cluster size (m) 45 and intra-cluster correlation coefficient (ICC) 0.02 [15]. The intra-cluster correlation coefficient value was calculated on the basis of cases of clinical respiratory illness in a previous cluster randomised clinical trial of personal protection masks [16]. The design effect (deff) for this cluster randomization trial was 1.88 (deff = 1+(m−1)×ICC = 1+(45−1)×0.02 = 1.88). As such, we aimed to recruit a sample size of 8 ACFs per arm.

### Random assignment of ACFs to oseltamivir use strategy

1. The ACFs were stratified by architecture type due to the possible influence of building design on infection transmission:
  - Type A: old dormitory style, multi-storey, non-purpose-built ACFs with communal toilets and bathrooms (making these facilities ‘more crowded’)
  - Type B: modern single-level purpose-built ACFs with adjoining assisted-living apartments (ALAs – low-level care independent living units) at the same site, with some shared facilities, staff and resident movements
  - Type C: modern single-level purpose-built ACFs without adjoining ALA
2. A third-party researcher assigned participating ACFs (within each stratum) to one of the oseltamivir use strategies by using a computerized stratified random process.

ACF and study staff were not blinded once random allocation was generated.

### Inclusion criteria for individuals

Any resident or staff member of a participating ACF was eligible to receive the intervention randomly assigned to the facility, provided individual written consent was obtained from the subject or the subject's guardian/legal representative (when dementia or other incapacity was present).

### Exclusion criteria for individuals

Exclusion criteria were known allergy to oseltamivir, symptoms of influenza for more than 48 hours, pregnancy, and end-stage renal disease or a creatinine clearance estimated to be 10 ml/min or less.

### Other influenza control strategies during the trial

The research team reminded each participating ACF to implement the influenza prevention measures recommended at the time by the Australian Government Department of Health and Ageing and New South Wales Health Department, for the prevention of influenza outbreaks in ACFs [17]. These measures include annual influenza vaccination of residents and staff, and standard infection control practices for respiratory infections (including hand washing, protective equipment and isolation), and restricting access to the facility.

### Definitions of: ILI, ILI outbreak and influenza outbreak

An ILI case was defined as acute onset of fever ≥38°C, with acute cough or any other respiratory sign or symptom in a resident or staff member. An ILI outbreak was defined as two ILI cases over a three day period, or, three ILI cases over a seven day period. An influenza outbreak was defined as an ILI outbreak with at least one ILI case having influenza virus detected by point of care testing, direct immunofluorescence or nucleic acid testing.

### Training of selected ACF staff in ILI surveillance and testing for influenza

Research team members visited participating ACFs in order to explain the study to staff. At each ACF, selected ACF staff members agreed to be ILI surveillance officers for that facility and were trained in active ILI surveillance and were provided with and trained in the use of the point of care testing [18].

### ILI surveillance

In order to identify as many ILI outbreaks as possible during the study period, a system of active influenza surveillance was instituted [18]. During influenza seasons study staff telephoned ACF ILI surveillance officers three times each week. At other times when influenza was not active in the community, the telephone calls were made once-weekly. At each call ACF ILI surveillance officers were asked to report the number of residents and staff who had ILI. If any ILI cases were reported at an ACF, the frequency with which study staff telephoned that facility increased to daily until either an ILI outbreak was identified or until 8 days had passed since the last ILI case.

### Identification of influenza outbreaks

ACF ILI surveillance officers performed influenza A and B specific point of care tests on ILI cases after consent had been obtained. If this identified influenza in a facility or if outbreaks of ILI were identified without a positive point of care test, the study team investigated the outbreak. The study team consisted of physicians, nurses and epidemiologists. This team performed an epidemiological investigation to determine the outbreak's aetiology, its spread and what control measures had to be taken. If an influenza outbreak was confirmed, residents and staff were treated from the date of declaration according to the oseltamivir usage policy assigned to that ACF, and the research team emphasised compliance with the infection control policy [17].

### Influenza case definitions

We defined a confirmed influenza case as an ILI case with identification of an influenza virus by point of care test, direct immunofluorescence or nucleic acid testing, culture or a ≥4-fold rise in complement fixing antibody titres. A probable influenza case was defined as an ILI case in the same floor or wing as a confirmed influenza case and who was either not tested for influenza or was tested but had negative results. A possible influenza case was defined as a person with respiratory signs and symptoms without fever who was in the same floor or wing as a confirmed influenza case and who was either not tested for influenza or was tested but had negative results.

### Identification of the ‘first case’ in each outbreak

We sought to identify the ‘first case’ in each ILI outbreak by establishing an apparent sequence of transmission. We identified all persons (staff or residents) who had respiratory symptoms and who were resident or working within an isolatable unit (e.g. the wing or floor of the ACF) in which an outbreak was occurring. Their dates of symptom onset were determined and ranked in date order. For influenza outbreaks, their symptoms were classified according to the probable and possible influenza case definitions and an apparent sequence of transmission was accepted when there was no more than three days between the onset dates of a probable influenza case and the previous probable influenza case.

### Data collected (influenza outbreaks)

Data collected for each consented subject was: the category of person who had ILI (i.e. staff versus resident), date of birth, age, sex, room occupied (resident) or main work area (staff) within the ACF, co-morbidities, medications, influenza vaccinations in the last 3 years, pneumococcal vaccination in the last 5 years, ILI onset date, measured body temperature, presence and duration of respiratory signs and symptoms, possible adverse events, hospitalisation details, pneumonia, death, general practitioner visits, plus data arising from diagnostic samples collected during the study.

### Diagnostic samples (influenza outbreaks)

When an influenza outbreak was declared, swabs were collected from the nose and throat of each consenting resident and staff member who either had ILI or was eligible to receive prophylaxis. The swabs were transported to the laboratory at 4°C in viral transport medium. In addition, acute and convalescent serum samples were collected 4 to 6 weeks apart.

### Laboratory methods

Nose and throat swabs samples were collected from individuals with ILI for point of care testing (QuickVue Influenza A+B Test; Quidel Corp., San Diego, CA., USA) [19]. Direct immunofluorescence was performed using cells from nose and throat swabs spotted on glass slides. The cells were acetone-fixed and stained with fluorescein-conjugated monoclonal antibodies (Chemicon International, Temecula, CA, USA) against influenza A and B and other respiratory viruses (adenoviruses, parainfluenzaviruses and respiratory syncytial virus) [20]. Nucleic acid testing was performed using a nested reverse transcriptase polymerase chain reaction for influenza A and B on RNA extracted from the nose and throat samples using the High Pure viral RNA kit (Roche Diagnostics GmbH, Mannheim, Germany) according to the manufacturer's instructions [21].

Influenza virus isolation (culture) was undertaken in MDCK cells. After 4 days incubation (35°C) the cells were stained with fluorescent influenza A & B monoclonal antibodies (SimulFluor FluA/FluB MoAb, Light Diagnostics, Temecula, CA, USA). Virus subtyping was performed by the WHO Collaborating Centre for Reference and Research on Influenza (Melbourne, Victoria, Australia) on a sub-set of isolates.

Influenza A- and influenza B-specific complement fixing antibody titres were determined on acute and convalescent sera in parallel, with definitive influenza recorded if there was a four-fold or greater rise in titres [20].

### Calculated values

In comparisons of baseline characteristics of ACF (Table 1), consented residents (Table 2) and staff (Table 3), we used (1) Two-sample t-test for continuous data with normal distribution, (2) Wilcoxon's Rank Sum Test for continuous variables with non-normal distribution, and (3) Two-sample Chi-square Test for binomial data. SPSS Version 19 (IBM, USA)[Computer Software] was used for these calculations.”

**Table 1:** Baseline characteristics of influenza outbreak aged care facilities by treatment allocation.

Baseline characteristic	T&P outbreak facilities (n = 6)	T outbreak facilities (n = 3)	Two-tailed p value
Mean number of residents	66	85	0.33
Mean number of beds in facility	86	89	0.91
Mean number of individual staff	58	72	0.51
Mean (median) time between ILI onset in the first case and declaration of outbreak (days)	5.0 (5)	12.7 (12)	0.06
Mean number of cases in residents prior to declaration of outbreak	5.7	12	0.08
Mean number of cases in staff prior to declaration of outbreak	4	5	0.67

**Table 2:** Baseline characteristics of consented residents in outbreak versus non-outbreak facilities.

	Consented residents with baseline data (n = 905)				
	Outbreak Facilities			*Non-Outbreak Facilities*	
	T	T&P	T versus P p value	Non-Outbreak facilities	Outbreak vs Non-outbreak p value
**Number**	77	314		514	
**Mean age**	80.7	81.5	0.51	81.8	0.08
**Female: Male**	1.7	2.4	0.17	1.6	0.11
**(n)**	(48:29)	(221:93)		(131:79)	
**Mean number of co-morbidities**	4.5	4.9	0.07	4.9	0.64
**Mean weight (kg)**	62.8	61.7	0.57	60.9	0.49
**Influenza immunisation received during the autumn vaccination period immediately preceding the influenza outbreak (if known) %**	83.6%	84.8%	0.95	81.1%	0.70
**(N)**	(46/55)	(251/296)		(340/419)	

**Table 3:** A comparison of the baseline characteristics of consented staff who were involved in the nine influenza outbreaks compared to those in facilities with no confirmed influenza outbreaks.

	Consented staff (no. = 275)				
	Outbreak Facilities			Non-outbreak facilities	
	T	T&P	T versus P p value	Non-Outbreak facilities	Outbreak vs Non-outbreak p value
**Number**	20	154		101	
**Mean age**	43.27	47.09	0.34	43.80	0.12
**Female: Male**	9.0	10.8	0.82	8.8	0.68
**(n)**	(18:2)	(140:13)		(88:10)	
**Mean number of co-morbidities**	2.20	1.70	0.11	1.94	0.61
**Influenza immunisation received during the autumn vaccination period immediately preceding the influenza outbreak (if known)**	50%	34.8%	0.41	27.4%	0.36
**(n)**	(9/18)	(49/141)		(17/62)	

As each influenza outbreak occurred in a different ACF, facility-level observations can be assumed to be independent. The primary outcome of attack rate was analysed using Poisson regression, with the count of number of influenza cases per facility regressed on treatment group, and the number of residents within each facility incorporated as an offset to allow for variation in facility size. Negative binomial regression was used where Poisson models showed greater than expected variability in counts arising from clustering. The secondary outcomes of attack rate in staff, resident deaths during outbreak and cases of chest infection and pneumonia were analysed in the same way. Due to problems with model convergence, hospitalisation of residents was analysed using the exact Wilcoxon test. Outbreak duration was also analysed using the exact Wilcoxon test. The randomisation test was used to obtain exact *p*-values [22].

### Mathematical modeling of influenza outbreaks

Mathematical modelling was undertaken to investigate the spread of influenza within ACFs under different assumptions of susceptibility. This had the advantage of providing sensitivity analyses for the main results. The model assumed a pool of susceptibles comprising all residents and staff of each ACF where an outbreak occurred. Before the epidemic, a proportion of the susceptibles are “removed”, by virtue of immunity from either prior vaccination or infection. The model assumes that individuals go through an incubation period of *d* days before they are recorded in the epidemic curve, such that individuals who are recorded as symptomatic on day *t* were infected on day *t*−*d*. Individuals develop both infectivity and symptoms following infection. The relative infectivity of each case was assumed to increase initially and then decrease so as to give a mean serial interval of 2.6 days. The expected number of infections on each day is calculated from the force of infection, taking into account the depletion of the pool of susceptibles.

Without intervention, the force of infection operating in an ACF on each day of an epidemic is determined from the number of people infected on each of the preceding days, multiplied by an unknown constant *θ*. In the period after intervention, *θ* is replaced with *θ*_T_ for T ACFs, and *θ*_TP_ for T&P ACFs. The three constants (*θ*, *θ*_T_ and *θ*_TP_) were determined by statistical analysis: a likelihood function was used to compare the expected number of infections on each day with the observed number of infections. A statistically significant difference between *θ* and *θ*_T_ or *θ*_TP_ indicates an effect of the intervention. The efficacies of the interventions were defined as 1−*θ*_T_/*θ* (for treatment only) and 1−*θ*_TP_/*θ* (for treatment & prophylaxis), so that an efficacy of 100% means that transmission is totally interrupted by the intervention.

The infectiousness and disease profile from Ferguson was adapted and simplified [23]. We assumed a mean incubation period of 1 day, and a mean serial interval of 2.6 days between cases. We considered there were too many uncertainties to build a model incorporating the prevalence of pre-existing strain specific immunity among residents or staff due to vaccination and previous exposure, so as a sensitivity analysis, we tested assumptions that that 50%, 75% or 100% of patients and staff were initially susceptible to influenza. For further sensitivity analysis, we re-analysed the data under assumptions that the incubation period was 0, 1 or 2 days, and that the serial interval was 2.2, 2.6 or 3.2 days.

## Results

**Figure 1:**
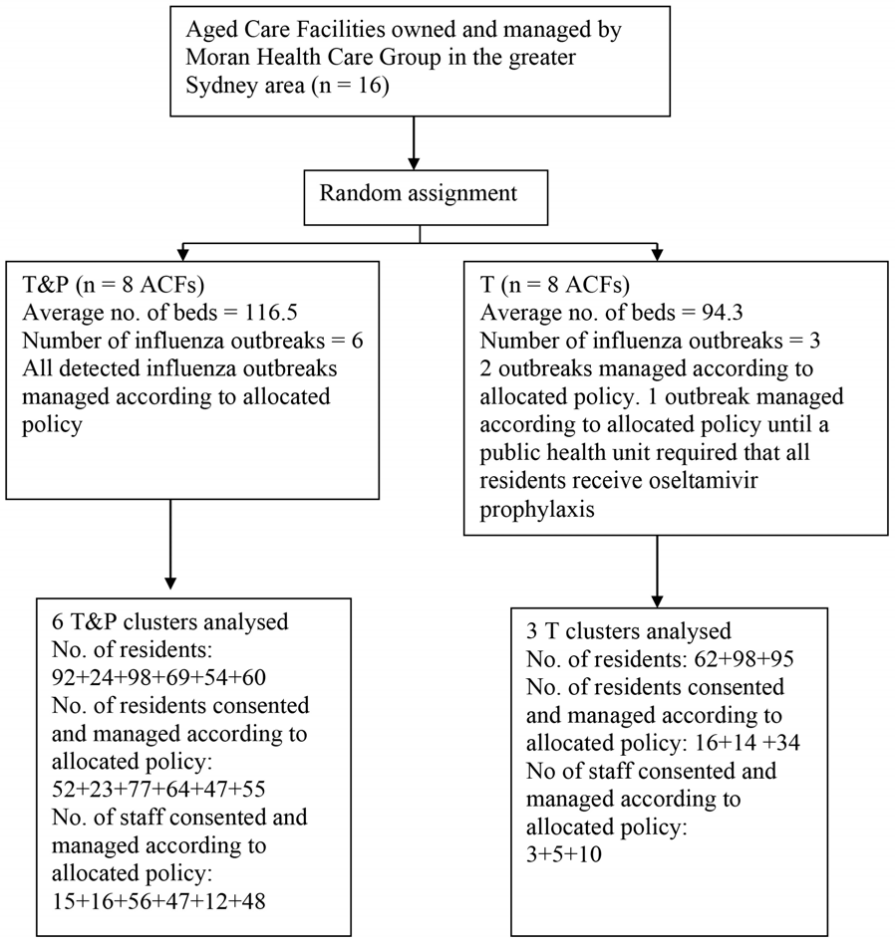
Flow diagram of progress of clusters and individuals in the trial.

We recruited 16 ACFs in the Greater Metropolitan Area of Sydney, Australia and the trial was carried out between 30 June 2006 and 23 December 2008, covering three southern hemisphere influenza seasons. The flow of participants is shown in Figure 1. The baseline characteristics of the ACFs with influenza outbreaks allocated T and those allocated T&P were not statistically significantly different (Table 1).

**Figure 2:**
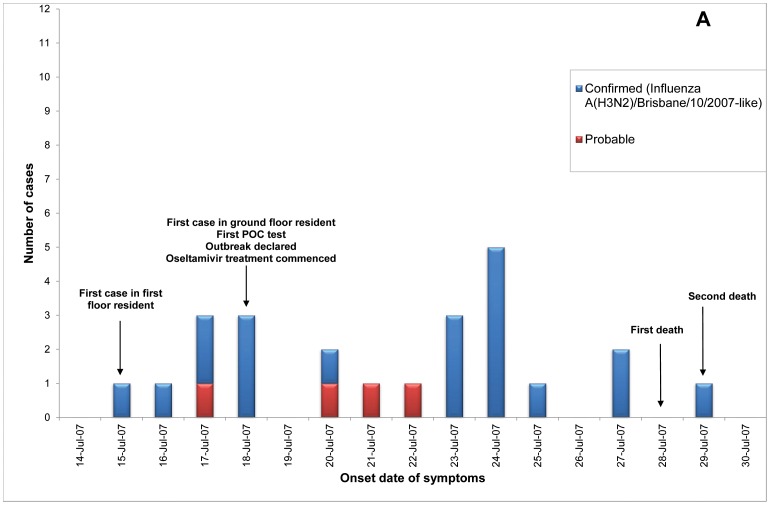
Epidemic curve for the influenza outbreak in Treatment only (“T”) Aged Care Facility A (confirmed and probable cases, amongst residents and staff).

**Figure 3:**
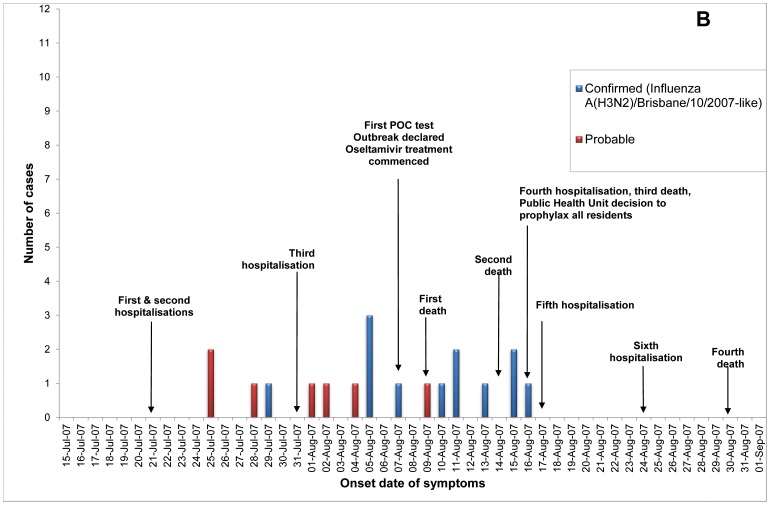
Epidemic curve for the influenza outbreak in Treatment only (“T”) Aged Care Facility B (confirmed and probable cases, amongst residents and staff).

**Figure 4:**
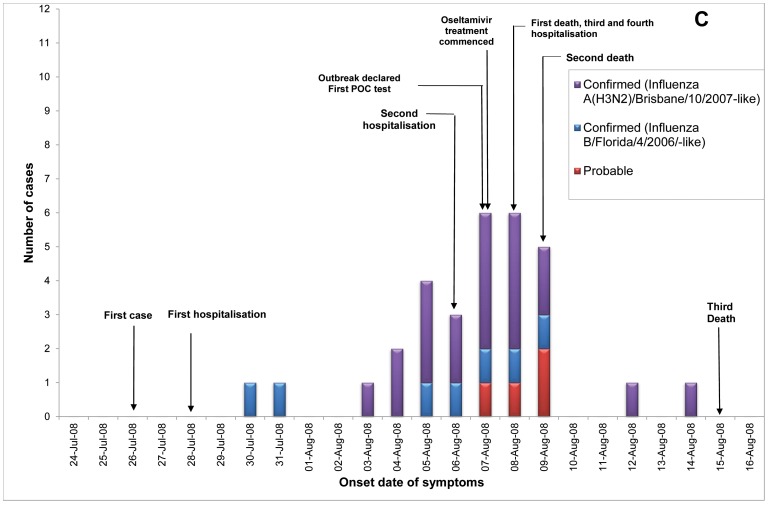
Epidemic curve for the influenza outbreaks in Treatment only (“T”) Aged Care Facility C (confirmed and probable cases, amongst residents and staff).

**Figure 5:**
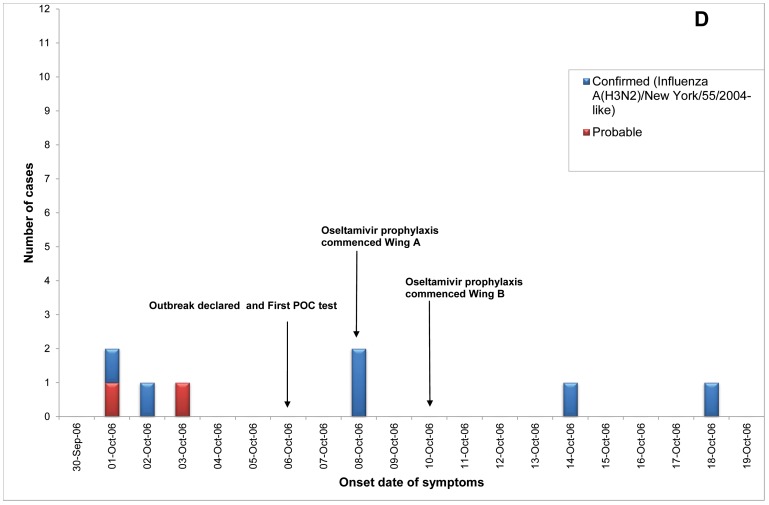
Epidemic curves for the influenza outbreak in Treatment and Prophylaxis (“T & P”) Aged Care Facility D (confirmed and probable cases, amongst residents & staff).

**Figure 6:**
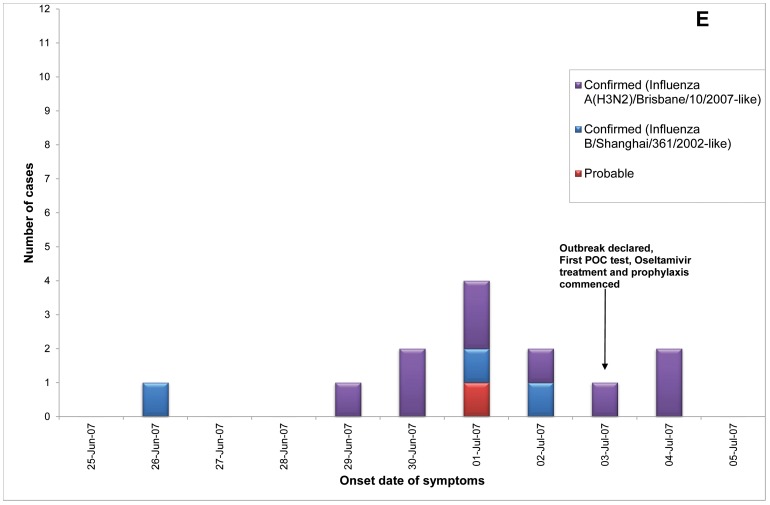
Epidemic curves for the influenza outbreak in Treatment and Prophylaxis (“T & P”) Aged Care Facility E (confirmed and probable cases, amongst residents & staff).

**Figure 7:**
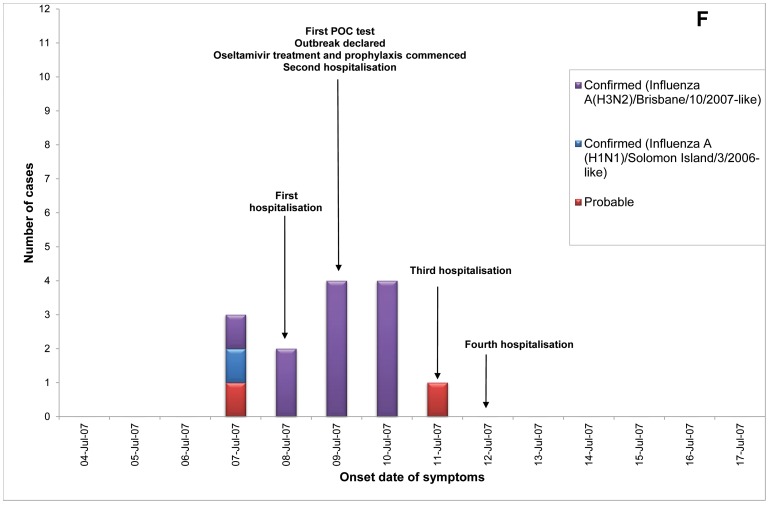
Epidemic curves for the influenza outbreak in Treatment and Prophylaxis (“T & P”) Aged Care Facility F (confirmed and probable cases, amongst residents & staff).

**Figure 8:**
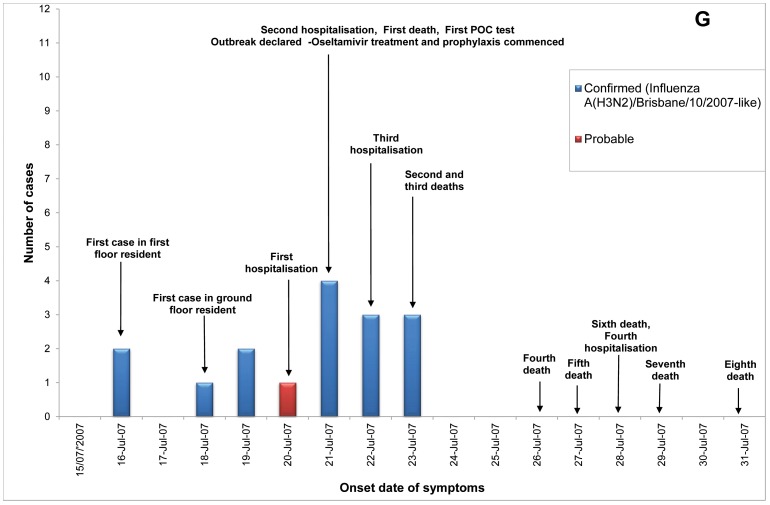
Epidemic curves for the influenza outbreak in Treatment and Prophylaxis (“T & P”) Aged Care Facility G (confirmed and probable cases, amongst residents & staff).

**Figure 9:**
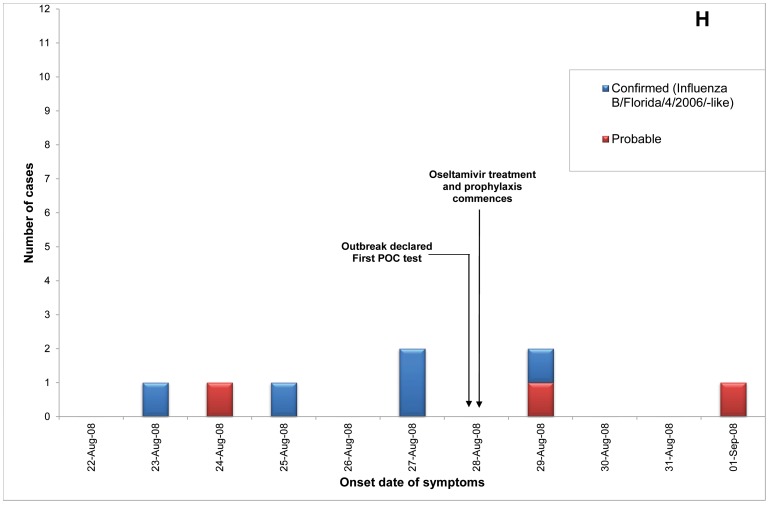
Epidemic curves for the influenza outbreak in Treatment and Prophylaxis (“T & P”) Aged Care Facility H (confirmed and probable cases, amongst residents & staff).

**Figure 10:**
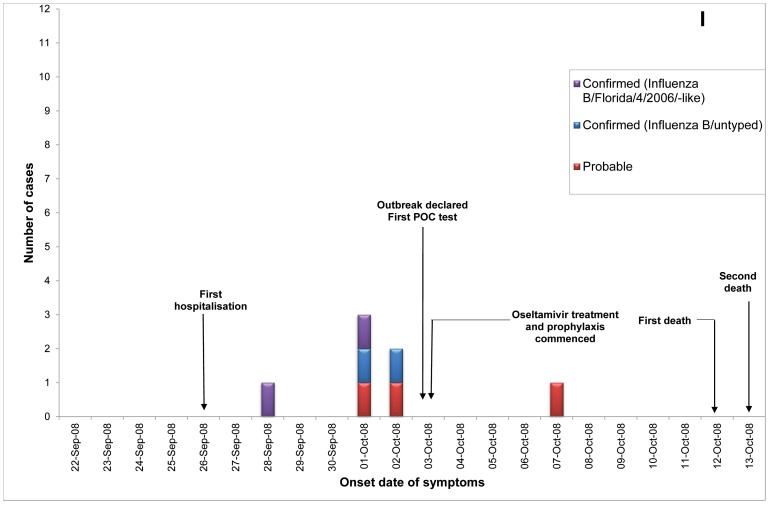
Epidemic curves for the influenza outbreak in Treatment and Prophylaxis (“T & P”) Aged Care Facility I (confirmed and probable cases, amongst residents & staff).

Of 23 ILI outbreaks identified: nine (39%) were due to influenza viruses; two (9%) were caused by respiratory syncytial virus; two (9%) by parainfluenzaviruses, one (4%) rhinovirus; and nine (39%) had no confirmed viral agent. Three of the influenza outbreaks occurred in T ACFs (Figures 2, 3, 4) and six in T&P facilities (Figures 5, 6, 7, 8, 9,10). The numbers of residents for each outbreak was determined by the layout and architecture of the facility and by the actual occupancy of the facility at the time of the outbreak.

Despite significant attempts to obtain consent from/for residents in advance of anticipated influenza seasons, consent usually had to be obtained during the influenza outbreaks. If an outbreak was identified in a T facility, for logistical reasons, only the residents and staff with ILI were approached for consent. However, in T&P facilities consent was required from/for anyone receiving treatment or prophylaxis. The baseline characteristics of consented individuals within each randomised facility were similar in the “T” and “T&P” facilities (Tables 2 and 3, Figure 1). During the actual outbreaks, in T&P facilities, 318 out of 397 (80%) of the residents and 194/350 (55%) of the staff consented to receive the oseltamivir to deliver the randomised policy. In T facilities 64 out of 255 (25%) of the residents and 18 out of 216 (8%) of the staff consented to receive oseltmivir in order to deliver the randomised policy. Of note, these ACFs had good coverage of influenza vaccination in the preceding autumn, with rates of 84% and 85% for “T” and “T&P” ACFs respectively.

Of the nine confirmed outbreaks, one occurred in 2006 (a T&P ACF), five occurred in 2007 (3 T&P ACFs; 2 T ACFs) which was a severe epidemic year [24], and three in 2008 (2 T&P and 1 T). The mean (median) time from onset of ILI in the first case to the declaration of an outbreak was 12.7 (12) and 5.0 (5) days respectively in T vs T&P ACFs (*p* = 0.06) and the mean number of cases in residents prior to declaration of the influenza outbreak was 12 in the T ACFs and 5.7 in the T&P ACFs, p = 0.08 (Table 1). The allocated policy was applied to all outbreaks but in ACF B (a treatment only allocated facility) the local Public Health Unit (PHU) commenced additional prophylaxis of residents late in the outbreak.

The mean duration of influenza outbreaks was 15.2 days (range 8 to 37); 10.8 days in the T&P ACFs compared to 24 days in the T ACFs (p = 0.03). Excluding the outbreak in ACF B (where the Public Heath Unit converted the treatment allocation into T&P), the mean outbreak duration of the 2 remaining T ACFs was 26 days.

**Figure 11:**
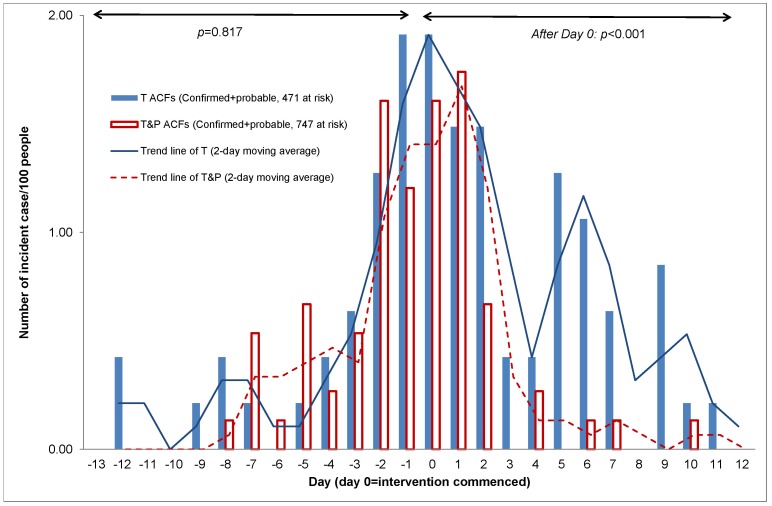
Number of incident cases (confirmed+probable) per 100 people (residents & staff) in T and T&P ACFs.

The observed attack rate in residents was significantly lower in T&P facilities compared to T facilities (Rate Ratio 0.63 95% Confidence Interval 0.47 to 0.84, p = 0.002), with an observed attack rate of 36.5% in T ACFs compared to 22.9% in T&P ACFs. The reduction in attack rate amongst staff was non-significant (Rate Ratio 0.71 95% CI 0.26 to 1.93, p = 0.5), with an attack rate of 21.3% versus 13.4%, respectively. In exploratory post hoc sensitivity analyses, the trial intervention was also significant when analysed by attack rates confined to confirmed and probable influenza cases (Figure 11). However, the results were insignificant when confined to confirmed influenza cases only. Confirmed influenza cases in residents were 19.6% (T only) versus 10.8% (T&P), p = 0.08; and in staff 3.2% (T only) versus 4.6% (T&P), p = 0.4.

We identified concurrent co-circulation of two different influenza viruses in 4 of the 9 outbreaks (see Table 4). The secondary outcomes of hospitalisation, death, pneumonia alone and pneumonia plus chest infection (of all types) in residents were all non-significantly reduced in the T&P facilities (Table 4), and irrespective of whether ACF B is included.

**Table 4:** Attack rates and viral types of identified outbreaks.

	Treatment only facilities (T)			Treatment and prophylaxis facilities (“T&P”)						Rate Ratio (95% CI); P
	ACF A	ACF B^1^	ACF C	ACF D	ACF E	ACF F	ACF G	ACF H	ACF I	
**Facility Type ** #	A	B	C	C	C	C	A	B	B	
**Duration of outbreak (days)**	15	37	20	17	11	11	8	9	9	P = 0.04^*^
**Attack rate in**	36.5% (93/255)			22.9% (91/397)						
**residents**	32.2% (20/62)	33.7% (33/98)	42.1% (40/95)	14.1% (13/92)	29.2% (7/24)	22.4% (22/98)	34.8% (24/69)	18.5% (10/54)	25.0% (15/60)	0.63 (0.47 to 0.84); P = 0.002+
**Number of residents treated with oseltamivir**	16	14	34	2	6	11	15	7	10	
**Number of residents prophylaxed with oseltamivir**	0	0	0	50	17	66	49	40	45	
**Attack rate in**	21.3% (46/216)			13.4% (47/350)						
**staff**	26.2% (11/42)	10.8% (9/83)	28.6% (26/91)	5.1% (4/79)	52.0% (13/25)	16.3% (15/92)	15.7% (8/51)	0.0% (0/26)	9.1% (7/77)	0.71 (0.26 to 1.93); P = 0.5†
**Number of staff treated with oseltamivir**	3	5	10	1	1	7	2	3	5	
**Number of staff prophylaxed with oseltamivir**	0	0	0	14	15	49	45	9	43	
**Hospitalisation rate of**	4.7% (12/255)			3.5% (14/397)						
**residents**	0% (0/62)	6.1% (6/98)	6.3% (6/95)	4.3% (4/92)	0.0% (0/24)	4.1% (4/98)	7.2% (5/69)	0% (0/54)	1.7% (1/60)	P = 0.7^*^
**Deaths during**	3.5% (9/255)			2.5% (10/397)						
**outbreak**	3.2% (2/62)	4.1% (4/98)	3.2% (3/95)	0.0% (0/92)	0.0% (0/24)	0.0% (0/98)	11.6% (8/69)	0.0% (0/54)	3.3% (2/60)	0.74 (0.12 to 4.48); P = 0.7†
**Case of chest infection (including pneumonia) reported**	0	4	5	0	1	0	6	1	1	0.78 (0.15 to 3.96); P = 0.8†
**Cases of pneumonia reported**	0	1	2	0	0	0	4	0	0	0.94 (0.07 to 11.79); P = 1.0†
**Outbreak virus(es)**	Influenza A(H3N2)/Brisbane/10/2007-like	Influenza A(H3N2)/Brisbane/10/2007-like	1) Influenza A(H3N2)/Brisbane/10/2007-like 2) Influenza B/Florida/4/2006/-like	Influenza A (H3N2)/New York/55/2004-like	1) Influenza A(H3N2)/Brisbane/10/2007-like 2) Influenza B/Shanghai/361/2002-like	1) Influenza A(H3N2)/Brisbane/10/2007-like 2) Influenza A (H1N1)/Solomon Island/3/2006-like	Influenza A(H3N2)/Brisbane/10/2007-like	Influenza B/Florida/4/2006/-like	1) Influenza B/Florida/4/2006/-like 2) Influenza B not subtyped	

# Type A: Old dormitory style; Type B: modern single-level purpose built facility with adjacent independent living unit; Type C: modern single-level purpose built facility.

Analysed using exact Wilcoxon test.

+ Analysed using Poisson regression.

† Analysed using negative binomial regression.

1 Late in the outbreak, the local public health unit started prophylaxis with oseltamivir for the remaining residents.

### Adverse drug reactions

None of the residents or staff stopped taking the prescribed oseltamivir due to adverse events. Adverse events were reported in 23.1% for those prescribed oseltamivir for treatment (data available for 108 residents) and 10.4% for those prescribed oseltamivir for prophylaxis (data available for 250 residents). Rates of adverse events for residents who received oseltamivir for treatment were: headache 7%; vomiting 5%; vertigo/dizziness 3% and nausea 2%. Rates for residents who took oseltamivir for prophylaxis were: headache 2%; vomiting 2%; vertigo/dizziness 2%; and nausea 3%.

Adverse events were reported in 76% of staff who took oseltamivir for treatment (data available from 21 staff) and 28% for those who took oseltamivir for prophylaxis (data available from 137 staff).

Adverse event rates for the staff who took oseltamivir for treatment were: headache 48%, nausea 29%, vertigo/dizziness 19% and vomiting 10%. Adverse event rates for staff who took oseltamivir for prophylaxis were: headache 15%, nausea 15%, vomiting 6% and vertigo/dizziness 4%.

### Mathematical modeling

Under the various assumptions investigated in the mathematical modeling, the T&P intervention strategy is predicted to reduce the number of transmission events attributable to each case by a significant amount. The T strategy was effective under the assumptions in the Base Case and also if a long serial interval or low degree of prior immunity was assumed. In every case, the median estimate of the efficacy of T was outside the 95% Confidence Interval of the efficacy for T&P, indicating that T&P was more effective for preventing transmission (Table 5).

**Table 5:** Mathematical modeling.

Assumed fraction with prior immunity	Strategy	Efficacy (% reduction) median estimate	Efficacy (95% credibility interval)
25%	Treatment	45	(23, 61)
	T & P	72	(56, 83)
50%	Treatment	30	(5, 50)
	T & P	71	(55,82)
55%	Treatment	21	(−7, 43)
	T & P	70	(53, 81)

Efficacy of Treatment only (“T”) vs Treatment and Prophylaxis (“T&P”) strategies for preventing transmission of influenza, assuming a latent period of 1 day and a serial interval of 2.5 days. Efficacy is expressed as the percentage of secondary cases prevented by the intervention.

“Fraction with prior immunity” is necessarily lower than fraction vaccinated, as the vaccine is not 100% effective and non-vaccine strains may be responsible for outbreaks. In our modeling, as 45% of ACF C residents were infected, our data can only be used to model up to 55% assumed fraction with prior immunity.

## Discussion

These results provide evidence that supports a policy of routine oseltamivir treatment and prophylaxis for residents and staff during proven influenza outbreaks in aged care facilities. Treatment and prophylaxis was associated with a reduced attack rate, and a non-significant reduction in secondary outcomes. Our results support recent guideline statements advocating such a policy [25]. Previously, Schilling et al demonstrated in a pilot study that zanamivir appeared promising when used in a large ACF in the United States, but to our knowledge further trials have not been attempted with this or other neuraminidase inhibitors [26].

Strengths of our study include the prospective cluster-randomised controlled trial design run over three influenza seasons. Our active surveillance and detailed epidemiological investigation of potential outbreaks led to far more influenza outbreaks being detected than usually reported in New South Wales. Our sample size assumptions proved rather optimistic given we stated that we expected to see attack rates of 10% (or 15%) attack rate in the T arm and 3% (or 6%) in the T&P arm. The higher than expected rates in both arms could have arisen for many different reasons: our detailed investigations might have revealed the true attack rates in ACF outbreaks; the style of nursing may have contributed as all our facilities were under the same management company; or perhaps our enhanced education and infection control sessions with facility staff were not as successful as we hoped. An important unanticipated result from our study was that influenza was only responsible for a minority of ILI outbreaks with 14 out of 23 respiratory illness outbreaks being due to other viruses (or no aetiological agent identified). More expansive nucleic acid testing may have identified the other viral causes of outbreaks; only direct immunofluorescence was used to detect a limited range of respiratory viruses as the interventions were directed to confirmed influenza outbreaks. Our results suggest that “Point of care testing” and laboratory confirmation of influenza or other respiratory virus infections is important in the investigation of ILI outbreaks in ACFs, and it is unwise to assume that all ILI outbreaks in ACFs are due to influenza.

The non-significant reduction in our secondary pre-specified outcome measures (deaths, hospitalisations and chest infections in residents) provide additional reassurance that the policy of treatment and prophylaxis is appropriate. These results was achieved despite our inability to treat all “at risk” residents and staff due to the constraints of the trial design (some declined consent) and the practicalities of treatment (some residents and staff were not eligible for treatment). The research design, including the requirement for individual informed consent possibly limited the proportion of residents and staff who accepted the interventions, and it is possible that greater coverage with oseltamivir prophylaxis would achieve greater protection.

The weaknesses of our trial were the low number of influenza outbreaks (nine) and the play of chance on randomisation, with influenza outbreaks occurring in three T ACFs and six T&P ACFs. Recruitment of more nursing homes, and thus clusters, would have helped balance randomization but this was not feasible given our funding and partnership with the ACF provider. The limited number of outbreaks reduced the power of the trial, especially for the secondary outcomes, which whilst generally in favour of the T&P policy, did not reach statistical significance. We recognise that our trial was underpowered as our main results were not confirmed in some of our sensitivity analyses, given the limited number of randomised facilities with outbreaks during the study. The imbalance of outbreaks amongst the two cluster randomised groups did not lead to statistically significant differences between the baseline variables of the outbreak characteristics or residents but the delay in declaring the outbreak in the T only facilities, and corresponding increase in the number of residents who were sick at the time of the implementation of the trial intervention could have contributed to the positive result of the trial. The imbalance in the number of residents and staff consented was due to the requirement of written informed consent for all residents who would be offered treatment. In T facility outbreaks, residents without symptoms did not need to be treated (or tested) and were therefore not approached for consent during an outbreak. In T&P facilities, we were required to obtain consent in all eligible residents in order to deliver the prophylactic arm of the trial. Despite attempts to consent all residents prior to outbreaks, the turnover of residents and huge workload required, meant that we were only able to consent a minority prior to an outbreak. Given that this differential consent rate could have potentially identified more people with subclinical influenza in the T&P facilities, we may have underestimated the effectiveness of a policy of T&P.

Our experience emphasizes the need for careful planning and investment to rapidly establish a treatment and prophylaxis intervention during outbreaks. In the trial, numerous extra research staff (up to ten) were enlisted during outbreaks yet it was often 2–3 days before it was possible to complete prophylaxis of all at-risk consented residents and staff. Some of the most time consuming duties may be lessened in routine clinical practice, such as a written consent process. However, other tasks would remain difficult to achieve quickly, such as assessing the renal function of every resident, and establishing whether they, or their legal guardians wished treatment to be given, unless this had been determined beforehand as recommended [25]. The lack of blinding in our study could have created bias in case ascertainment, but a double-blind study would probably be unfeasible, or at least prohibitively expensive.

Our mathematical modeling of transmission also showed a substantial reduction in the reproduction number in the T&P facilities, relative to the T facilities. This reduction is due to decreased susceptibility of prophylaxed patients, and decreased infectivity of breakthrough and undetected cases. The basic reproduction number for influenza is assumed to be 2 (or less) in the general population although it may be much higher in modified communities such as ACFs. After allowing for some prior immunity and vaccination, it seems plausible that a reduction in transmission of 70% would be required to explain the containment of the epidemics in the T&P group.

We acknowledge the difficulty of conducting trials in ACFs, and, in retrospect, the resources required to undertake the trial were underestimated. As a result of our unexpected difficulties in establishing the intervention, some of the planned data collection was not possible. Given the low power of our study, we believe that further research to confirm these results would be important.

What are the implications of this study for routine public health policy? We believe that public health departments should explore the feasibility and benefits of introducing an active surveillance approach for ILI in ACFs during at risk periods such as winter. This could be done by telephone or electronic communication, and centralised for a large number of nursing facilities. The potential advantage of early diagnosis and treatment may not only prevent unpleasant symptoms and illness for residents but also for their carers, including staff and relatives. Laboratory confirmation of outbreaks will also allow a clearer understanding of the clinical features and outcomes of non-influenza virus outbreaks in the frail elderly. Our observation of multiple viral outbreaks occurring simultaneously within an ACF and the concurrent illness of staff suggest that ACFs provide an important focus of viral infection in winter months and a possible mechanism of maintaining infection in the community. Even if the potential benefits to residents of reducing the burden of illness is discounted, control of infection in ACFs may be an important public health intervention for the wider community. Although deaths amongst younger people such as the staff of ACFs are rare, they have been reported [2]. We would argue that infection control, including antiviral treatment, is important.

Our results need to be considered in the light of reports of oseltamivir resistance, and increased resistance will clearly attenuate the effectiveness of a T&P policy [27]. As the more widespread use of prophylaxis could increase resistance, surveillance for influenza A subtypes and oseltamivir resistance in local geographic areas is important [28]. On the other hand, our results provide indirect support that the use of other antiviral medication for treatment AND prophylaxis might be an effective policy for ACFs.

An important point is that data on drug efficacy and safety is rarely available for the frail vulnerable patient: this trial is a step forward in providing some evidence for those in ACFs. With our ageing populations, many millions of people are cared for in such institutions and it is important to establish the risks and benefits of interventions for this population who are usually excluded in medical research. We appreciate the difficulties in conducting trials for such populations but we hope that others will also consider studies involving the frail older resident of ACFs.

## Supporting Information

Checklist S1 **CONSORT Checklist.**
(DOC)

Protocol S1 **Trial Protocol.**
(PDF)
