# Supplementary material for: Treating and Preventing Influenza in Aged Care Facilities: A Cluster Randomised Controlled Trial
Source: PLoS One. 2012 Oct 17;7(10):e46509. doi: 10.1371/journal.pone.0046509 (PMC3474842; doi:10.1371/journal.pone.0046509)
Supplement: Protocol S1 — Trial Protocol. (PDF) [file pone.0046509.s002.pdf]

The University of Sydney

NSW 2006 Australia

Human Research Ethics Committee
www.usyd.edu.au/ethics/human
Manager:
Gail Briody
Telephone: (02) 9351 4474
Facsimile: (02) 9351 6706
Email: gbriody@usyd.edu.au
Rooms L4.14 & L6.04 Main Quadrangle A14

Human Secretariat
Telephone: (02) 9036 9309
(02) 9036 9308
Facsimile: (02) 9036 9310

23 November 2007

Professor Robert Booy
Director, Division of Research
National Centre for Immunisation Research and
Surveillance of Vaccine Preventable Diseases
The Children's Hospital at Westmead – C29

Dear Professor Booy

Title: ***Medical, economic and social benefits of treating and preventing influenza in aged care facilities (pilot study 2005 for formal intervention study 2006-2008)***

Ref: ***8446***

Thank you for providing the signed copy of the Memorandum of Understanding, fulfilling the condition of approval as stated as stated in your modification approval letter dated 29 October 2007 and the revised version of the protocol [12/11/2007].

Yours sincerely

Professor D I Cook
Chairman
Human Research Ethics Committee

Encl.
Protocol [12/11/2007]

Economic and Social Benefits of Treating and Preventing Influenza in Aged Care Facilities (ACFs)

Investigators
Prof Robert Booy
A/Prof Raina MacIntyre
Dr Dominic Dwyer
Prof Richard Lindley

UNIVERSITY OF SYDNEY
HUMAN ETHICS COMMITTEE
APPROVED
DATE: 21 NOV 2007

UNIVERSITY OF SYDNEY
HUMAN ETHICS COMMITTEE
APPROVED
DATE: 21 NOV 2007

Aims:
1. To assess the value of the anti-influenza drug Tamiflu as treatment only, compared with its use in both treatment and prevention for residents of ACFs.
2. To collect data on the emergence of drug resistance to Tamiflu.
3. To model the best use of Tamiflu when planning for influenza outbreaks in:
   A. aged care facilities, projecting for the ageing Australian population
   B. the likely forthcoming global pandemic of avian influenza

Background
Influenza is a cause of significant morbidity and mortality, particularly in the elderly during the annual winter epidemics (1). Diagnosed influenza, which represents the "tip of the iceberg", causes more morbidity and mortality than any other infectious disease in Australia; with the highest hospitalisation rates for influenza at the extremes of age. The virus can cause primary viral pneumonia, or secondary bacterial pneumonia, and causes higher mortality in persons with underlying chronic diseases (1;2). The vaccine is efficacious in preventing influenza, and in reducing the incidence of pneumonia and death in the elderly. Influenza immunisation is a cost-effective intervention, and expert committees around the world recommend it for people over the age of 65 and for those with predisposing medical risk factors. The National Health and Medical Research Council (NHMRC) of Australia recommend that adults aged 65 years and over should be immunised with both influenza and pneumococcal vaccines (3). Other persons who have medical risk factors are also recommended for immunisation. However, vaccination rates in eligible hospitalised inpatients are suboptimal.
Although immunisation against influenza is recommended for high-risk populations in Australia including those aged over 65 years, it is less effective in the elderly, and an added benefit is possible by using antiviral medication. Further, over the last few years, there have been vaccine shortages due to industrial production problems. As such, other approaches are crucial. An under-utilised but highly effective intervention is the antiviral drug oseltamivir (Tamiflu), a neuraminidase inhibitor.

The ageing of the Australian population – importance of geriatric medicine
As our population ages an increasing acute hospital workload results from the management and assessment of frail elderly patients. During the influenza season, nursing home residents contribute significantly to geriatric hospital admissions. In New South Wales there are approximately 900,000 people aged 65 years and older and they account for 500,000 hospital separations each year (4). There are approximately 80,000 elderly people residing in ACFs in Australia, with this number projected to rise to 200,000 by 2040, when an estimated 25% of the population will be aged 65 years or older. This increase will be driven by the dramatic increase in the number of people aged 85 years and older which will rise from about 300,000 people to some 1.1 million by 2042. The chance of being in a nursing home rises exponentially with age, being less than 1% for those aged 65 to 74 years old, 3.5% for the 75-84 year olds and 15% for the 85 years and over (5). Whilst we hope there will be further compression (postponement to later in life) of morbidity and that this future aged population will be healthier than their preceding cohort, we cannot guarantee that this will be the case. We therefore need to plan accordingly. Even with the most optimistic forecasts, there is no doubt that the absolute numbers of people in nursing home accommodation will continue to increase in Australia for the foreseeable future. With these trends, an ageing workforce is predicted, with the average age of retirement increasing. The long-term social implications of aged care and related health issues are significant, with two of the stated priorities of the

HUMAN ETHICS COMMITTEE
APPROVED
DATE: 21 NOV 2007

National Strategy for an Ageing Australia (Minister of Health 2002) being "A strong evidence base should inform the policy responses to population ageing", and "A care-system that has an appropriate focus on the health and care needs of older Australians and adequate infrastructure to meets needs".

Pandemic influenza
The recently established endemicity of H5N1 influenza in birds in South-East Asia is a serious threat to human health. This is due to the risk of the emergence of a pandemic strain following reassortment of avian and human virus strains or multiple mutations in the avian H5N1 strain (6). A pandemic strain to which the population is immunologically naïve would result in considerable morbidity and mortality, and would affect proportionately younger people compared to seasonal epidemic influenza. In recognition of the serious nature of an influenza pandemic, Australia has a National Pandemic Influenza Plan. Yet there are still gaps in knowledge about the transmission dynamics of influenza and its impact on both annual epidemic and pandemic planning. Without understanding both the direct and indirect consequences of influenza, this impact cannot be estimated. Of major concern are the impact of a pandemic on acute health services, and the capacity of our health and aged care systems in such an emergency. Current pandemic planning includes stockpiling of oseltamivir, but there are few data on the optimal approach to antiviral treatment in different populations (7). Key questions are whether the best approach is to treat cases only, or to also offer prophylaxis to case contacts or a particular mix of the two. Possible limiting factors are the development of anti-viral resistance, but there are no good data to inform policy decisions. An understanding of both the direct and indirect consequences of using antivirals to treat and prevent influenza will aid population disease control policy.

Institutional influenza outbreaks – relevance to pandemic planning
Transmission of influenza within closed institutions is a recognised problem, and one which results in significant morbidity and costs (8-10). The high risk of transmission of influenza in hospitals, for example, is demonstrated by the example of a hospital acquired outbreak in Spain that occurred in the absence of a community epidemic of influenza (11).
Understanding the transmission dynamics of institutional influenza is important for pandemic planning, as well as for planning of aged care. In the event of a pandemic, treatment and management in institutions may act to amplify disease transmission, much as they did during the SARS outbreak (12). Influenza, however, is far more infectious than SARS, with high levels of virus in respiratory secretions much earlier in the course, so that spread would be even more rapid and nursing home infection control even more crucial. The role of hand-washing and other infection-control practices has not been studied extensively in this context. It is known that compliance with hand-washing, even in teaching hospital settings, is less than 50% (13). During the SARS epidemic, infection control practices were shown to be crucial to disease control, (12;14) but their importance and relative impact compared to vaccination and antiviral use in an influenza epidemic is unknown.

Role of vaccination and antivirals
We know from institutional outbreaks of influenza that prior vaccination of staff and patients is clearly protective (9;15). In addition, neuraminidase inhibitors (NI) are available for the treatment and prophylaxis of influenza A and B infections (16;17). In acute influenza, if started within 24 - 48 hours of onset, they can dramatically reduce the length and severity of disease. In households with an index case of influenza, prophylaxis for 10 days reduces transmission to others by about 80% (16). NIs can also be used as prophylaxis in high risk groups during the influenza season (e.g. for 6 weeks). NIs can also be used to reduce the

impact of outbreaks in ACFs (18). However, the optimal use of this drug as prophylaxis, treatment or a mixture of both is unknown, as both modes of use have not been trialled head to head. This gap in knowledge is what our study aims to address. The oral NI oseltamivir is licensed in Australia for use in the treatment and prophylaxis of influenza. Resistance to the NIs has been reported to be uncommon in clinical trials, although resistance (in a single study) was more common in children treated with oseltamivir (19). There is no data on the frequency of NI resistance in the elderly.

The use of a clinical case definition of influenza
Case definitions for surveillance of "influenza-like-illness" (ILI) are used internationally but with considerable variation. In Australia, the simplest definition used is presentation with fever, cough and fatigue (20). Some jurisdictions use the definition of sudden onset plus four of the following: (21)

- fever,
- cough,
- rigours or chills,
- prostration and weakness,
- myalgia,
- influenza in close contacts,
- no significant respiratory signs other than redness of the nasal mucous membranes and throat.

UNIVERSITY OF SYDNEY HUMAN ETHICS COMMITTEE
APPROVED
DATE: 21 NOV 2007

A clinical case definition of ILI is crucial in a pandemic or epidemic for surveillance and rapid estimates of vaccine effectiveness, when time or circumstances do not permit timely laboratory diagnosis. A recent study of involving Hajj pilgrims (22) demonstrated that a combination of subjective fever, sore throat and cough was a specific, sensitive and simple case definition for use in the setting of mass gatherings. The positive predictive value of ILI is much higher in an epidemic than during a non-epidemic period, so that it is likely to be a very useful case definition for surveillance during a true pandemic. However, there are few available data which validate ILI with diagnostic evidence, and hence the use of ILI is often dismissed by purists as inadequate. This study will enable the test characteristics (sensitivity, specificity, positive predictive value, negative predictive value) of the common ILI case definitions to be measured.

Memorandum of understanding with NSW Health
A memorandum of understanding (MOU) has been established between the study investigators, the Communicable Diseases Branch of NSW Health and the Directors of Public Health Units (PHUs) within which the participating ACFs are located.

Study group responsibilities as per MOU (attached)
In the event of an influenza outbreak:

- The study coordinator, or other nominated contact, will notify the PHU responsible for the affected ACF, directly by phone within 24 hours, of any laboratory confirmed case of influenza. This includes cases diagnosed following rapid antigen detection testing.
- The study coordinator, or other nominated contact, will advise the PHU of the study arm (treatment alone *vs* treatment plus prophylaxis) to which the ACF has been randomly allocated.
- The study group will encourage and facilitate the participating ACF to implement or enhance recommended infection control measures for the management of an influenza outbreak.

- The study group will notify and consult with the relevant PHU if an outbreak within a treatment-alone ACF appears to be poorly contained despite early identification and treatment of cases. The PHU may modify the intervention allocated to the affected ACF if, in the opinion of the PHU Director, the health of the public is threatened.
- The study coordinator, at the request of the responsible PHU, will provide basic epidemiologic information regarding an outbreak, including the number of clinical cases, number of lab-diagnosed cases, number of participants taking treatment and number taking prophylaxis, possible links between identified cases, and the extent of compliance of participants to the treatment or prophylaxis protocol.
- The study group will not disclose individual identifying information to a PHU unless legally required or following specific consent being granted to do so from study participants or their guardians.

ACF responsibilities during an influenza outbreak
The study will not remove the responsibilities of individual ACFs in working with their respective PHUs during an influenza outbreak. The study group has no authority to direct participating ACFs to adopt or modify any specific infection control measures. If the ACF is unsure how to implement or interpret outbreak control measures they should contact their respective PHU for clarification. During an outbreak the ACF is responsible for providing a daily line listing to their respective PHU as per NSW Health guidelines.

GP Involvement
The study group will seek assistance from ACF staff in advising residents' treating GPs of any influenza outbreak that involves residents under their care. A prepared fax will be sent to GPs advising of an outbreak and asking them to contact the ACF or study group for further clarification. If fax details are unavailable ACF staff should contact GPs directly.

In the case of residents where no response to the initial letter of invitation has been received, those residents without a next-of-kin, or who have next-of-kin who are not readily contactable, the treating GP will be approached by a study group medical officer and be asked to prescribe oseltamivir for that resident. The study group medical officer will provide instructions regarding treatment and prophylaxis doses and a supply of oseltamivir will be left at the facility for the treating GP.

To avoid unnecessary delay in the commencement of therapy, study group medical officers may offer to prescribe on behalf of any GP who is unable to attend to a resident. To avoid unnecessary delay in the commencement of therapy, study group medical officers may offer to prescribe on behalf of any GP who is unable to or delayed in attending a resident.

Methods:
Study design: A cluster-randomised controlled trial of oseltamivir for control of influenza in ACFs.
Study population:

Subjects: Any person residing in a study ACF, or any staff member or volunteer working in a study ACF will be eligible. The ACFs will be 16 Moran Health Care Group ACFs in the greater Sydney metropolitan area. Moran Health Care Group, an aged care conglomerate, is the named industry partner on a successful ARC linkage grant # LP0668279, commenced in 2006. The unit of randomisation will be at the level of the ACF, with each facility randomised to either arm 1 or 2 (see below).

UNIVERSITY OF SYDNEY
HUMAN ETHICS COMMITTEE
APPROVED
DATE: 21 NOV 2007

Justification of clustering: Influenza is a communicable disease which once present may be transmitted throughout an individual ACF. In addition, use of Tamiflu may result in "herd effects" beyond the individual and as such the unit of randomisation needs to be the ACF.

Exclusion criteria:

1. Subjects who have a contraindication to Oseltamivir (known hypersensitivity to any component of the drug. Components are: Pregelatinised maize starch, talc, povidone K 30, croscarmellose sodium and sodium stearylfumarate. The capsule shell contains gelatin, titanium dioxide, iron oxide red CI77491, iron oxide yellow CI77492, iron oxide black CI77499, shellac and indigo carmine CI73015.
2. Subjects who are unwilling to give consent or in whom next of kin consent is not obtained. Guardianship Board approval will be sought to allow next of kin consent for people who cannot give informed consent themselves. The investigators have extensive experience in this area, having obtained guardianship board approval for other research involving demented patients.
3. Residents, staff or volunteers who have had symptoms suggestive of influenza for more than 48 hours will be ineligible to receive treatment with oseltamivir, as there is no evidence for efficacy if given after this time.
4. Staff who are pregnant or currently breastfeeding will be ineligible for oseltamivir.

Study drug

Oseltamivir is already registered in Australia for the approved indication of treatment and prophylaxis of influenza. The drug is already established as safe and effective, and used routinely to manage outbreaks by NSW Health. Our study is designed to determine the most effective way to use this drug, as treatment, prophylaxis or a combination of both. The drug will be supplied by Roche Australia. As both arms receive the same drug, and blinding is not possible because of the nature of the intervention (treatment vs prophylaxis and treatment) the standard labels which appear on the commercially available product will be used.

Dosage:

Arm 1: Oseltamivir for treating cases of influenza (defined by the clinical definition of "influenza-like illness" – ILI) (75 mg twice daily for 5 days)

Arm 2: Oseltamivir will be used for treating cases of influenza (75 mg twice daily for 5 days) in all staff and residents, in addition to prophylaxis (75mg once daily for 10 days).to all contacts in the nursing home.

Renal impairment and dosage adjustment:

Subjects with renal impairment (defined as an estimated creatinine clearance between 10-30 mmol/L) as determined by a review of their medical history or case notes will receive a half dose of oseltamivir for the prescribed period.

Randomisation procedure: Nursing homes will be randomly allocated to the two arms by a secure computer program. ACFs will be randomly assigned to receive antiviral prophylaxis with oseltamivir offered to all cases (defined as ILI after one confirmed case) and contacts (once a case is diagnosed), or antiviral treatment of diagnosed cases only and no antivirals for contacts. For cases, treatment is only indicated if given ≤48 hours of onset of symptoms (17).

UNIVERSITY OF SYDNEY
HUMAN ETHICS COMMITTEE
APPROVED
DATE: 21 NOV 2007

Definitions:

ACFs - Nursing Homes (NHs) and Hostels

ILI - sudden onset plus four of the following: fever, cough, rigours or chills, prostration and weakness, myalgia, influenza in close contacts, no significant respiratory signs other than redness of the nasal mucous membranes and throat. However because of the absence of classical respiratory signs and symptoms in some elderly persons (borne out by data from the first year of the study, 2006), indicators such as poor appetite and sudden behavioural change may also be used to assist in identification of potential cases in the setting of a proven influenza outbreak.

Staff – all employees (including volunteers) who may work within the ACF

Resident – any person living in the ACF

Contact – refers to someone who has had close contact with a person who has had influenza. For staff this refers to episodes such as provision of clinical care, feeding and toileting (generally within 1 metre of the case). For residents this may include sharing a room with a known or suspected case, or some other regular social interaction (e.g. dining with or visiting) a known or suspected case.

Treatment – therapy for residents and staff with proven influenza, ILI.or other clinically suspicious changes in the setting of an influenza outbreak

Prophylaxis – preventive therapy for asymptomatic residents and staff in the setting of an influenza outbreak

Relief medications:

There will be no restrictions on use of medications for symptom relief (such as paracetamol) for study subjects. The pharmacokinetic data on oseltamivir suggest that drug interactions are very unlikely. Relief medications for symptoms of ILI will be provided by the ACFs. If relief medication is required for symptoms arising from the use of oseltamivir, this will be covered by the study budget. The most likely symptom is nausea. However, given the dangers of polypharmacy in geriatric patients, and the risk of neuropsychiatric side effects with anti-emetics, each case will be reviewed individually before anti-emetics are given.

Assessment of clinical symptoms and potential adverse events:

A data collection form ("yellow") will be inserted into the medication charts of all residents receiving treatment or prophylaxis to enable ACF staff to document symptoms of ILI and potentially any symptoms related to the administration of oseltamivir. The same data collection form will also be provided to staff to collect details of their illness and any potential side-effects from antiviral medication usage.

UNIVERSITY OF SYDNEY
HUMAN ETHICS COMMITTEE
APPROVED
DATE: 21 NOV 2007

Characterisation of the study cohort:

The cohort of ACFs will be characterised by:

- the number of residents, whether bedrooms are shared, and ~~the size and extent (time-wise)~~ of social interaction in common rooms
- the age-range and degree of disability (physical and mental) of residents the number of staff, type of staff, what protocols exist regarding infection control and how well staff are aware of these

- whether there are annual campaigns of influenza vaccination, records kept of these and opportunistic vaccination to new arrivals
- the turnover rate of residents and whether policy and practice of vaccination exists, whether a log is kept of visitors from outside, including their relationship to the resident, and whether they have an acute illness (especially respiratory) and have been vaccinated against influenza

Qualitative research will be carried out before the winter with staff and residents to address issues of acceptability and feasibility. Semi-structured interviews and focus groups will be used to inform the study. This project will involve the training of nurses in infection control, disease surveillance, health promotion including vaccination, testing for influenza and treating with drugs. These nurses in turn will be given training in how to train other nurses and allied professionals. This will assist with influenza outbreak control but also more generally in keeping the elderly well and safe from other transmissible infections causing respiratory or gastrointestinal disease.

Regular staff surveys will be done. This will be used to analyse the spread of influenza within the ACF, with a focus on the roles of ACF crowding, early isolation, hand washing, vaccination (patients and staff), use of antivirals and other infection control procedures. A staff survey will be conducted during the same time period, recording staff movements within the ACF, influenza vaccination history and infection control practices. These data will also be analysed as predictors of incident infection.

Active disease surveillance:

ACF staff will be asked to monitor for ILI in residents and among each other. When two or more cases of ILI occur within three days, or three cases occur with in a seven day period this will be deemed a respiratory outbreak. ACF staff are to contact the study team who will undertake an assessment of risk and the need for further investigation. If the outbreak specifics include fever ≥38°C accompanied by a cough in two or more residents the study teateam will wherever possible attempt to collect nose and throat swabs and acute sera for respiratory virus testing in any consented resident.

Other triggers for investigation of outbreaks

The study team will attempt wherever possible to investigate any clusters of respiratory illness identified to them during surveillance, including those where a febrile illness has not been reported. This is important given the absence of classical signs and symptoms (or poor recognition of such) in many residents with proven influenza infections. Swabs and / or blood will be collected where possible from consented residents and staff. The decision to undertake investigation of clusters of ill residents will be left to the discretion of the study co-ordinator in consultation with the primary investigators.

To assist with establishing a rapid influenza diagnosis each ACF will be regularly and amply supplied with point-of-care (POC) influenza antigen detection kits. ACF staff are encouraged to use these. If any single resident or staff member has a positive POC test, this will result in an influenza outbreak being declared and prophylaxis and/or treatment being offered. A negative POC test does not exclude influenza as the cause of the respiratory illness outbreak.

Baseline study data:

UNIVERSITY OF SYDNEY
HUMAN ETHICS COMMITTEE
APPROVED
DATE: 21 NOV 2007

Baseline data to be collected for residents and staff include: standard socio-demographic and clinical data (including age, gender, influenza and pneumococcal vaccination history, current medications, co-morbidities etc). For each subject, an influenza status will be established and the presence or absence of influenza symptoms (as described above under "clinical case definition of ILI") will be recorded.

Outcome measurements:

The primary outcomes to be measured include:

1. Incidence of influenza as measured by
   a. ILI
   b. Laboratory confirmation
2. Death/case fatality rates
3. Pneumonia (both clinical diagnoses and those confirmed by CXR)
4. Hospital admission
5. Attack rate per nursing home in staff and residents
6. Staff absenteeism
7. Development of oseltamivir resistant influenza
8. Adverse events following oseltamivir

Management of Adverse Events

Information on serious adverse events (SAEs) will be collected throughout the trial by members of the study team. These will then be assessed by the chief investigators and reported on using the SAE Initial and SAE Follow-up Reporting forms. Updating will be undertaken if relevant information becomes available later (e.g., outcome, medical history, results of investigations, copy of hospitalization reports). The chief investigators will assess any causal relationship between the SAE and oselatmivir use.

Sample size:

Power calculations: a reduction in case fatality rate (attack rate) from (30) 10% to (10) 3% (Or 15% to 5%) over a winter season would be easily achieved (power >80%) with the involvement of 16 ACFs each with an average of 45 residents. We will also look at time to cessation of outbreak as an outcome. Intra-ACF correlations (IAC) will also be calculated to explore any effects due to clustering. For each outcome, if the intra-cluster correlation (ICC) values are very small, this will indicate that clustering has a negligible effect on P values.

Collection and testing of samples

Testing of two types of specimens will be done. These are nasal and throat swabs and blood samples. This testing will be undertaken for both residents and staff who are receiving treatment, in addition to those receiving prophylaxis. Study team staff will accord priority to the collection of specimens from symptomatic consented residents and staff. This is to ensure that these persons are treated as promptly as possible.

UNIVERSITY OF SYDNEY
HUMAN ETHICS COMMITTEE
APPROVED
DATE: 21 NOV 2007

UNIVERSITY OF SYDNEY
HUMAN ETHICS COMMITTEE
APPROVED
DATE: 21 NOV 2007

Blood samples: Serum samples will be taken from consented residents and staff with symptoms of influenza at onset and then 4-8 weeks later to determine rise in influenza-specific antibody titres.

Nasopharyngeal samples: Nasal swabs and throat swabs will be taken as soon as possible following the onset of symptoms. They will again be taken three days later (Day 3) and six days later (Day 6) until resolution of symptoms or until a negative immunofluorescence result for influenza is documented.

In circumstances where consented residents are unable to have specimens collected, for example following a transfer to hospital, the study team will make efforts to have specimens collected externally and forwarded to ICPMR for standard testing.

Laboratory testing for influenza

Sample collection: A swab will be collected by a trained staff member, usually a nurse, epidemiologist or doctor. The 'collector' will follow standard clinical procedure. Specimens will be transported as soon as possible after collection to the laboratory, or at 4°C if transport will be delayed (e.g. overnight). Specimens collected on a weekend, including a Friday, may require storage in an -80°C freezer to ensure preservation.

Testing:
CI Dr Dwyer's laboratory CIDMLS is a WHO National Influenza Centre, and in NSW provides state-wide reference services for respiratory viruses. Although unlikely to be required, PCR methods have been developed for avian influenza, and BSL3 level facilities are available.

1. Diagnosis of influenza (or other respiratory viruses) will be performed as soon as possible on specimens collected from symptomatic residents or staff in the setting of a confirmed or suspected influenza outbreak. Methods used will include direct immunofluorescence assay (DIF), PCR (23) and/or serology, using standardised laboratory methods. Influenza virus isolates will be further serotyped using hemagglutination inhibition using WHO supplied antibodies.

2. Testing for drug resistance
This will be undertaken if the initial clinical specimen is positive for influenza following testing by DIF or PCR and subject has been treated with oseltamivir. Nose and throat swabs will be collected at Day 3 and Day 6 or every three days until the resolution of respiratory symptoms or until DIF or PCR becomes negative for influenza. Isolates will be stored for referral to the WHO Collaborating Centre for Reference and Research on Influenza for phenotypic resistance studies (Professor Ann Kelso and Dr Ian Barr have agreed to collaborate) (24). Genotypic resistance studies for oseltamivir mutations will be undertaken according to published methods (25).

Nucleotide sequencing:
Sequencing of influenza virus genomes will be undertaken using specific primers in the HA1 domain of hemagglutinin of H3N2 and H1N1 as used in previous studies (26) to determine the relatedness between different viral isolates (thus helping determine the origin of viruses within the outbreaks). CI Robert Booy has previous experience with this type of analysis, and has been able to describe genetic homology in influenza viruses detected within (but not

between) families (27). These results are consistent with a previous demonstration of identical HA1 nucleotide sequences recovered from families infected with influenza (28). CI Dominic Dwyer has experience with this approach in influenza outbreaks in closed environments such as ACFs and prisons (18;29).

Timelines: The study will run for three years. This timeframe is subject to some variability, depending on the onset of winter influenza activity (as the study is dependant on influenza activity being present). The study period will commence after three confirmed cases of influenza are identified by the hospital laboratory. It is anticipated this will be some time between July and September (generally the peak months of influenza activity in Australia).

Analysis:
Descriptive analysis, including epidemic curves for individual ACF's and for the two influenza management arms will be performed. Rates of influenza and of drug resistance, as well as the impact of influenza outbreaks on factors such as staff absenteeism, resident hospitalisation and death will be described. Rates of ACF-transmitted influenza will be compared between intervention and control ACFs, adjusting for rates of influenza at baseline, influenza vaccination status and other socio-demographic and clinical confounders. Multivariate logistic regression analysis will be done to determine predictors of influenza, focusing on vaccination status and use of antivirals as key predictor variables. Evidence of emerging NI-resistant strains of influenza A will be sought as well as whether the use of antiviral prophylaxis and/or treatment is associated with such drug resistance.

Mathematical and economic modelling
In most sciences, research questions are answered by planned repeated experiments. For infectious diseases experimenting in communities is rarely ethical or possible. Instead, we rely on observational data that are not timely. Infectious diseases are unique because mutually exclusive states of immunity, infection and susceptibility exist in humans, enabling us to quantify the transmission dynamics of diseases within populations. Mathematical models which incorporate characteristics of organisms and of the population can predict the nature and timing of epidemics, the spread of organisms throughout a population and can be used to compare the effectiveness of disease control strategies.
We lack adequate data to develop and evaluate response plans for pandemic influenza. Australia currently relies on expert judgement in the management of infectious disease threats due to the absence of adequate relevant data. There is large uncertainty as to the potential effectiveness of interventions. Modelling is an invaluable tool allowing study of disease behaviour in the absence of contemporary experience with the disease. Modelling can be used to assess risk and identify populations or areas of increased risk, and the effectiveness of control strategies. Modelling methods used will include both stochastic and dynamic approaches and be based on a number of relevant variables including the spatial distribution of the population at risk, the effective contact rate, and surveillance and control activities. Parameters of the models will be varied to estimate the effects of different scenarios such as points of introduction, or surveillance and control activities, including vaccination, antivirals or delayed recognition.
Matrices of age-specific social contact patterns of nursing home residents, staff and their outside contacts will be developed and refined. Stochastic and dynamic mathematical models will be developed to quantify the transmission dynamics and impact of interventions on annual and pandemic influenza. Data collected from the study on the impact of oseltamivir, vaccination and infection control on influenza incidence, transmission and drug resistance will be used to develop mathematical models to predict the most effective use of these control

HUMAN ETHICS COMMITTEE
APPROVED
DATE: 21 NOV 2007

strategies in aged care facilities and during a pandemic. Once the mathematical modelling is completed, cost-effectiveness and cost-utility analyses of the use of oseltamivir for cases only compared to cases and contacts will be conducted. Direct and indirect costs will be included. Surveillance, mortality and hospitalisation data from 1993-2005 will be used to determine the overall age-specific incidence of influenza, deaths and hospitalisations. Direct medical costs, per case, will estimated by disease stage and frailty. Data collected during the study will be used to estimate the cost of outbreak control and of absenteeism. The price of oseltamivir per course will be based on the wholesale price. The assumed protective efficacy of oseltamivir in the two scenarios will be based on observed efficacy in the study, as well as published data. A discount rate of 5% will be applied to costs and benefits. Quality of life estimates will be derived by a pragmatic application of the EuroQol-5D (EQ-5D) instrument (30).

Access to data

All data will be stored on computer disc and as paper records within the National Centre for Immunisation Research in accordance with NSW Department of Health regulations. Computer discs will be erased and paper records shredded after a minimum period of 15 years. In all respects confidentiality of medical records will be kept.

Storage and disposal of data

The investigator must keep all trial documents for at least 15 years after the completion or discontinuation, whatever the nature of the investigational centre (private practice, hospital, and institution).

Subjects' names will be coded using initials and a study number. This is to protect his/her privacy when the information collected during the study is computerised and used by the investigator.

Significance statement

This work will provide essential real data on the best use of a life-saving drug that has already been stockpiled by the government (over 3 million courses) in the event of a pandemic. It can determine how to get best value – who to use the drug in, whether to give it just for treatment or prevention as well and how soon drug resistance is likely to become a problem. The project will collect novel information on a disease which has two major areas of significance:

1. Planning for pandemic influenza
2. Planning for annual influenza in the context of ACFs and in the context of projecting future needs for an ageing Australian population.

The innovation is two-fold:

1. A novel investigation that addresses a gap in international knowledge concerning use of oseltamivir in various situations, and associated drug resistance.
2. A unique collaboration between public health and infectious diseases experts, with the participation of the largest nursing home conglomerate in Australia.

Economic benefits

1. Resident illness: considerable costs are accrued when ACF residents become ill. If hospitalisation is required, as is often the case, costs rise exponentially. During outbreaks of influenza in Australian ACFs, facilities must cease intake of new residents to prevent disease spread, thereby incurring considerable financial loss. The benefits of widespread use of oseltamivir in ACFs in preventing severe illness and hospitalisation, as well as closure of ACFs, will be significant.

HUMAN ETHICS COMMITTEE
APPROVED
DATE: 21 NOV 2007

2. Staff absenteeism accounts for a considerable proportion of economic loss in Australia. This is particularly so in the winter months. Although one day may be lost through sickness attributed to "the flu", when an employee truly has an influenza infection the likelihood is such that one week's productivity can easily be lost due to the severity of illness. Staff may be infected by their own children or by contact with other staff or residents. Time off may also result from the need to care for children or aged relatives not in care.
Optimising the size of facilities and their economic efficiency can, in part, be addressed through an analysis of disease and absenteeism rates by size of ACFs, resident staff ratios and mixing patterns. With the continued ageing of the Australian population, control of influenza in ACFs will become increasingly important.

UNIVERSITY OF SYDNEY
HUMAN ETHICS COMMITTEE
APPROVED
DATE: 21 NOV 2007

Reference List

(1) Nicholson KG, Wood JM, Zambon M. Influenza. Lancet 2003 November 22;362(9397):1733-45.

(2) Stephenson I, Zambon M. The epidemiology of influenza. Occup Med (Lond) 2002 August;52(5):241-7.

(3) Australian Technical Advisory Group on Immunisation (ATAGI). The Australian Immunisation Handbook. 8th ed. Canberra: Commonwealth Departmnet of Health and Ageing; 2007.

(4) Anonymous. Older People. New South Wales 2004. Canberra: Australian Bureau of Statistics; 2007.

(5) Drabsch T. Ageing in Australia. NSW Parliamentary Library Research Service; 2007. Report No.: Paper No. 12/04.

(6) Reid AH, Taubenberger JK. The origin of the 1918 pandemic influenza virus: a continuing enigma. J Gen Virol 2003 September;84(Pt 9):2285-92.

(7) Biosecurity and Disease Control Branch. National Pandemic Influenza Plan. Canberra: Australian Department of Health and Ageing; 2007.

(8) Barlow G, Nathwani D. Nosocomial influenza infection. Lancet 2000 April 1;355(9210):1187.

(9) Berg HF, Van Gendt J, Rimmelzwaan GF, Peeters MF, Van Keulen P. Nosocomial influenza infection among post-influenza-vaccinated patients with severe pulmonary diseases. J Infect 2003 February;46(2):129-32.

(10) Sartor C, Zandotti C, Romain F, Jacomo V, Simon S, Atlan-Gepner C et al. Disruption of services in an internal medicine unit due to a nosocomial influenza outbreak. Infect Control Hosp Epidemiol 2002 October;23(10):615-9.

(11) Horcajada JP, Pumarola T, Martinez JA, Tapias G, Bayas JM, de la PM et al. A nosocomial outbreak of influenza during a period without influenza epidemic activity. Eur Respir J 2003 February;21(2):303-7.

(12) Ho PL, Tang XP, Seto WH. SARS: hospital infection control and admission strategies. Respirology 2003 November;8 Suppl:S41-S45.

(13) Pittet D, Mourouga P, Perneger TV. Compliance with handwashing in a teaching hospital. Infection Control Program. Ann Intern Med 1999 January 19;130(2):126-30.

(14) Riley S, Fraser C, Donnelly CA, Ghani AC, Abu-Raddad LJ, Hedley AJ et al. Transmission dynamics of the etiological agent of SARS in Hong Kong: impact of public health interventions. Science 2003 June 20;300(5627):1961-6.

(15) Meiklejohn G, Hall H. Unusual outbreak of influenza A in a Wyoming nursing home. J Am Geriatr Soc 1987 August;35(8):742-6.

(16) Hayden FG, Atmar RL, Schilling M, Johnson C, Poretz D, Paar D et al. Use of the selective oral neuraminidase inhibitor oseltamivir to prevent influenza. N Engl J Med 1999 October 28;341(18):1336-43.

(17) Treanor JJ, Hayden FG, Vrooman PS, Barbarash R, Bettis R, Riff D et al. Efficacy and safety of the oral neuraminidase inhibitor oseltamivir in treating acute influenza: a randomized controlled trial. US Oral Neuraminidase Study Group. JAMA 2000 February 23;283(8):1016-24.

(18) Bush KA, McAnulty J, McPhie K, Reynolds R, Boomer M, Clarkson LM et al. Antiviral prophylaxis in the management of an influenza outbreak in an aged care facility. Commun Dis Intell 2004;28(3):396-400.

(19) Centers for Disease Control and Prevention. Prevention and control of influenza: recommendations of the Advisory Committee on Immunization Practices (ACIP). Morbidity Mortality Weekly Review 52[RR-8]. 2007.
Ref Type: Journal (Full)

(20) Thursky K, Cordova SP, Smith D, Kelly H. Working towards a simple case definition for influenza surveillance. J Clin Virol 2003 July;27(2):170-9.

(21) Clothier HJ, Fielding JE, Kelly HA. An evaluation of the Australian Sentinel Practice Research Network (ASPREN) surveillance for influenza-like illness. Commun Dis Intell 2005;29(3):231-47.

(22) Rashid H, Shafi S, Bashir HE, Haworth E, Memish ZA, Ali KA et al. Influenza and the Hajj: defining influenza-like illness clinically. Int J Infect Dis 2007 May 29.

(23) Druce J, Tran T, Kelly H, Kaye M, Chibo D, Kostecki R et al. Laboratory diagnosis and surveillance of human respiratory viruses by PCR in Victoria, Australia, 2002-2003. J Med Virol 2005 January;75(1):122-9.

(24) Hurt AC, Barr IG, Durrant CJ, Shaw RP, Sjogren HM, Hampson AW. Surveillance for neuraminidase inhibitor resistance in human influenza viruses from Australia. Commun Dis Intell 2003;27(4):542-7.

(25) McKimm-Breschkin J, Trivedi T, Hampson A, Hay A, Klimov A, Tashiro M et al. Neuraminidase sequence analysis and susceptibilities of influenza virus clinical isolates to zanamivir and oseltamivir. Antimicrob Agents Chemother 2003 July;47(7):2264-72.

UNIVERSITY OF SYDNEY
HUMAN ETHICS COMMITTEE
APPROVED
DATE: 21 NOV 2007

(26) Ellis JS, Chakraverty P, Clewley JP. Genetic and antigenic variation in the haemagglutinin of recently circulating human influenza A (H3N2) viruses in the United Kingdom. Arch Virol 1995;140(11):1889-904.

(27) Teo SS, Ellis JS, Aitken C, Booy R. Transmission of influenza A in families. Pediatr Infect Dis J 2007 July;26(7):645-7.

(28) Gubareva LV, Novikov DV, Hayden FG. Assessment of hemagglutinin sequence heterogeneity during influenza virus transmission in families. J Infect Dis 2002 December 1;186(11):1575-81.

(29) Young LC, Dwyer DE, Harris M, Guse Z, Noel V, Levy MH. Summer outbreak of respiratory disease in an Australian prison due to an influenza A/Fujian/411/2002(H3N2)-like virus. Epidemiol Infect 2005 February;133(1):107-12.

(30) EuroQol BIOMED research programme. The measurement and evaluation of health status using EQ-5D: A European perspective. London: Kluwer Academic Publishers; 2007.

UNIVERSITY OF SYDNEY
HUMAN ETHICS COMMITTEE
APPROVED
DATE: 21 NOV 2007
